# Supplementary material for: Content-rich biological network constructed by mining PubMed abstracts
Source: BMC Bioinformatics. 2004 Oct 8;5:147. doi: 10.1186/1471-2105-5-147 (PMC528731; doi:10.1186/1471-2105-5-147)
Supplement: Additional File 2 — The original results of the above study (non-essential files are deleted to keep the file size under the limit set by BMC bioinformatics). [file 1471-2105-5-147-S2.bz2 › chilibotAdditionalFile2/dip10/678ID7876093E11085/html/TNFRSF1A_TRAP1.html]

 


 **TNFRSF1A** and **TRAP1** 
  
Found 10 abstracts in PubMed, retrieved 10.  
 

 What does Google say? 
 PDF only 
| .edu only 

---

**Interactive relationship** (e.g. stimulation, inhibition, etc)

**Neutral relationship**- By using truncated TNFR  [ **TNFRSF1A** ]  2ICs for in vitro precipitation of 35S TRAP  [ **TRAP1** ] , it was shown that the C terminal half of the TNFR  [ **TNFRSF1A** ]  2IC contains the domain necessary for interaction with TRAP  [ **TRAP1** ] .  Ref: 7639698 Biochem J, 1995
- After in vitro transcription translation and 35S labeling, TRAP 1  [ **TRAP1** ]  was precipitated using a fusion protein consisting of glutathione S transferase and TNFR  [ **TNFRSF1A** ]  1IC, showing that the two proteins directly interact.  Ref: 7876093 J Biol Chem, 1995
- We have used the yeast interaction trap  [ **TRAP1** ]  to isolate a protein, MADD, that associates with the death domain of TNFR1  [ **TNFRSF1A** ]  through its own C terminal death domain.  Ref: 9115275 J Biol Chem, 1997

**Non-interactive relationship** (e.g. studied together, co-existance, homology, etc.)

- The ability of deletion mutants of TNFR 1  [ **TNFRSF1A** ]  to interact with TRAP 1  [ **TRAP1** ]  was tested using the two hybrid system.  Ref: 7876093 J Biol Chem, 1995
- After in vitro transcription translation and 35S labelling the TRAP  [ **TRAP1** ]  was precipitated using a fusion protein consisting of glutathione S transferase and the intracellular domain of TNFR  [ **TNFRSF1A** ]  2 TNFR  [ **TNFRSF1A** ]  2IC, which showed that the two proteins directly interact in a mammalian cell free system and also that identification of the TRAP  [ **TRAP1** ]  was not an artifact of the two hybrid system.  Ref: 7639698 Biochem J, 1995
- By combined use of the yeast interaction trap  [ **TRAP1** ]  system and a peptide scanning library, the novel WD repeat protein FAN has been identified, which specifically binds to a cytoplasmic nine amino acid binding motif of TNF R55  [ **TNFRSF1A** ] .  Ref: 8808629 Cell, 1996
- Identification of TRAP 1  [ **TRAP1** ]  may be an important step toward defining how TNFR 1  [ **TNFRSF1A** ] , which does not contain protein tyrosine kinase activity, transmits its message to signal transduction pathways.  Ref: 7876093 J Biol Chem, 1995
- The TRAP  [ **TRAP1** ]  identified in the present study shares considerable homology with, and may be the human homologue of, a mouse protein, TNF receptor associated factor 2 TRAF2, that binds mouse TNFR  [ **TNFRSF1A** ]  2.  Ref: 7639698 Biochem J, 1995
- In addition, using the yeast interaction trap  [ **TRAP1** ]  system, we have identified one protein FAN that binds to the NSD and mediates activation of N SMase as well as seventeen other potential interaction partners of TNF R55  [ **TNFRSF1A** ] .  Ref: 8913930 J Inflamm< MedlineTA>J Inflamm, 1995
